# On What Do the Homeless Base Their Happiness?

**DOI:** 10.3390/healthcare9111512

**Published:** 2021-11-05

**Authors:** Yaiza Cano-González, Carmen Portillo-Sotelo, María del Mar Rodríguez-del-Águila, María Paz García-Caro, Ana M. Núñez-Negrillo, Carmen Herrera-Espiñeira

**Affiliations:** 1 Andalusian Health Service (Servicio Andaluz de Salud), 18001 Granada, Spain; yaiza_cano@hotmail.com (Y.C.-G.); carmenporti18@gmail.com (C.P.-S.); 2 Preventive Medicine and Public Health Service (Servicio de Medicina Preventiva y Salud Pública), Hospital Universitario Virgen de las Nieves, 18014 Granada, Spain; mmar.rodriguez.sspa@juntadeandalucia.es; 3 Instituto de Investigación Biosanitaria ibs, 18012 Granada, Spain; cherrera_1@ugr.es; 4 Department of Nursing, Faculty of Health Sciences, University of Granada, 18016 Granada, Spain; mpazgc@ugr.es; 5 Research Network of Healthcare Services in Chronic Diseases (Red de Investigación de Servicios de Salud en Enfermedades Crónicas [REDISSEC]), 18012 Granada, Spain

**Keywords:** homeless persons, happiness, personal satisfaction, anxiety, depression, qualitative research

## Abstract

Objective: To determine the relationship between the characteristics and experiences of homeless persons and their state of happiness as a basis for designing appropriate social support strategies. Design: Exploratory observational study with an analytical and descriptive qualitative design. Setting: Participants were contacted, administered with questionnaires, and interviewed in the street (central and northern areas of the city) or at the “*Asociación Calor y Café*” center in Granada (Spain) between April 2017 and February 2018. Participants: Selected by intentional sampling, 25 participants completed questionnaires in the first study and 14 of these were administered with questionnaires and interviewed in the second study. Method: General and specific questionnaires were administered to determine the state of happiness and other variables. Descriptive statistics were followed by an analysis of the relationships between variables and the content analysis of semi-structured interviews. Results: A feeling of happiness was described by 64% of participants and confirmed by a happiness scale score of 50%. Participants who felt satisfied with their life were 4.5-fold more likely to feel happy (*p* = 0.021). Expectations for the future were not associated with happiness or satisfaction with life. Content analysis of interviews revealed three main themes: conditions for happiness, own happiness/unhappiness, and self-esteem. Conclusions: Many homeless people describe themselves as feeling happy and satisfied with their life. Material aspects, affective situations, daily life concerns, and self-esteem predominate in their discourse on happiness.

## 1. Background

The Federation of National Organizations Working with the Homeless (FEANTSA) [1] defines homeless persons (HPs) as those unable to achieve or maintain adequate and permanent accommodation adapted to their situation, either due to economic reasons, social barriers, or the inability to lead an autonomous life.

In Spain, the National Statistics Institute (INE) [2] reported that around 23,000 HPs visited social support centers in 2012, although it is estimated that more than 30,000 HPs in the country were not included in these figures [3]. In 2020, a mean of 17.772 HPs/day were accommodated by social services, 1.3% fewer than in 2018 [4].

In the study by Fazel, Geddes, and Kushel [5] on the health of HPs in high-income countries, homelessness was related to poverty, family problems, mental health issues, substance abuse, and/or structural factors such as the lack of low-cost homes. Little research has been conducted on more subjective aspects such as the general happiness or wellbeing of HPs, which may be useful to support the design of more effective interventions [6]. Ahuja et al. [7] found that their subjective well-being was inferior to that of people with homes. The health conditions of HPs pose a major care challenge to nurses working in the community, and an approach that accounts for HPs’ own perception of wellbeing and happiness may improve the effectiveness of interventions in this population, such as those that focus on happiness [8,9].

Obtaining information about and analyzing happiness in general is a highly complex task. Easterlin [10] considered that subjective indicators are useful to evaluate happiness, but the optimal approach remains under debate. A study of subjective well-being in 11 nations found that it was correlated with their social, economic, and cultural characteristics [11]. Layard [12] studied happiness in the general populations of 50 countries and concluded that it was influenced by seven main factors: family relationships, economic status, work, community, friendships, personal freedom, and personal values. All except for the economic factor refer to relationships. The quality and stability of relationships and concern for others have been reported to generate greater happiness in individuals than concern for themselves [13], leading to greater success in life [14],

One characteristic of HPs is that they vary in almost all the specific vital controls. In a study of 235 HPs in Madrid (Spain), 46.8% reported feeling happy, and a general state of happiness was associated with: not feeling alone or abandoned, not suffering disability or severe or chronic disease, having good expectations for the future, identifying with some religious belief, and having a positive perception of their own health status [15]. However, a study of 20 HPs in Australia found that health contributed little to their general perception of subjective wellbeing, which was more closely associated with feeling safe, being positive, feeling good, connecting with others, and participating in “normal” life [16]. A study of the characteristics of HPs in Granada (southern Spain), conducted by the present researchers in 2017, found that 6 of the 15 HPs then interviewed had been living on the street for more than eight years, and that 9 of them felt happy, a surprising finding given their difficult life conditions [17]. These observations, and the contradictory findings in the literature, prompted the development of the present investigation in the same city, using questionnaires and interviews to gain knowledge of the most important concerns of HPs, the reasons for their happiness or unhappiness, and their expectations for the future and satisfaction with their life, as expressed in their own words. The aim was to improve the relationship between HPs and care professionals, enhancing the trust needed for effective health care interventions.

The present study was comprised of two phases. The objective of the first phase was to determine the characteristics of HPs in Granada and to analyze the relationship between these characteristics and their self-perceived happiness. The objective of the second phase was to explore the factors that HPs considered to affect their happiness or unhappiness.

## 2. Methods

### 2.1. Design

In the first phase, an observational analytical study was conducted to determine the characteristics of HPs, their happiness status, and other study variables, using questionnaires. In the second phase, a qualitative descriptive study was carried out to explore the experiences of HPs using a content analysis of semi-structured interviews. The study was conducted between April 2017 and February 2018 in Granada (Spain).

### 2.2. Sampling

The enrolment of participants started after a two-year period (2016–2018) in which the principal researcher and other concerned individuals made regular nocturnal visits to places where people were sleeping on the street in the city of Granada, making contact with the homeless. Workers in the *Asociación Calor y Café* (Heat and Coffee Association) participated in these visits and became involved in the present project. This association runs a center used by many HPs in Granada that provides breakfast and an afternoon snack, facilities to wash and clean clothes, and leisure activities, as well as social support. Before the start of the present study, the researchers spent a large amount of time at this center to gain the trust and confidence of the users.

Participants in this study were HPs who visited the *Calor y Café* center or were found in the streets of the city. An intentional consecutive non-randomized sampling procedure was conducted that took account of no other characteristics, selecting 25 HPs who attended the *Calor y Café* center and consented to study participation and the audio recording of interviews. Exclusion criteria were signs of drunkenness and/or mental confusion at the time of the interview. A nocturnal survey of HPs in Granada in 2019 found 62 people sleeping on the street, without counting those sleeping in caves or abandoned buildings; the majority were aged between 46 and 50 years, and 89.3% were men [18].

### 2.3. Data Collection

The data were collected by two researchers working separately, who approached HPs in the street or while they were in the *Calor y Café* center for breakfast or an afternoon snack. After receiving an explanation of the purpose of the study and its voluntary character, their consent was sought for the completion of questionnaires and an audio-recorded interview. They were informed that all data would be treated anonymously in accordance with national and European data protection legislation (Spanish Law 15/1999, 13 December; EU regulation 2016/679, 27 April 2016).

In the first phase of the study, the questionnaire included the sociodemographic variables: age (years), years on the street, number of children, sex, origin (Andalusia, rest of Spain, foreigners), educational status (no studies/primary school, secondary school, professional training, university degree), causes of homelessness (economic crisis, divorce, family conflicts, other causes), health (good health, poor health), whether participant had been assaulted (yes, no), where the participant sleeps (on the street, in temporary lodgings), source of income (no income, non-contributory pension, temporary employment), jobseeker (yes, no), requested assistance from social services (yes, no), and whether he/she feels discriminated against (yes, no) and feels happy (yes, no).

After an interval of from two to three months, the above participants were contacted, when possible, for the second phase of the study, which was conducted at the *Calor y Café* center by the same researchers. The following questionnaires were administered:

- Diener’s Satisfaction with Life Scale (SWLS): This instrument has been validated in Spain [19] del instrument original [20]. It includes five questions on the responder’s overall satisfaction with life, with Likert-type responses ranging from 1 (completely disagree) to 5 (completely agree).
- General Happiness Scale: Visual scale evaluating general happiness in seven categories, represented by pictures of expressive faces: *very happy, rather happy, somewhat happy, neither happy nor unhappy, somewhat unhappy, rather unhappy, and very unhappy.* Participants respond to the question: *Which of the following faces best represents your level of general happiness?* This scale was used in the study by Vazquez et al. [21]
- Goldberg’s Anxiety and Depression Scale [22], using the version adapted to Spanish by Montón, Pérez Echeverría, Campos, García Campayo, and Lobo [23]. This has two subscales, one for anxiety and the other for depression, classifying responders as having or not having “*probable anxiety*” (cutoff: ≥4) and/or “*probable depression*” (cutoff: ≥2).
- A further question was added regarding their expectations for the future (improve/remain the same/worsen).

After completing the above questionnaires, a semi-structured interview was conducted, audio-recorded and transcribed. All participants were asked “*What is happiness for you?*”; if the response was positive, they were asked: *“What makes you happy*?”, and if negative, they were asked “*What would you need to feel happy?*”. Next, they were asked “*What time of your life do you remember as happiest?*” and, finally, “*Do you feel good about yourself*?”.

Interviews were individual, private, face-to-face, and of variable duration according to the situation. The researchers were previously trained to ensure an unprejudiced neutral attitude towards the homeless. The digital recording of each interview was downloaded in a password-protected computer and transcribed by the same researcher.

The study complied with EU regulations (2016/679) and Spanish legislation (3/2018) on personal data protection and digital rights and was conducted in accordance with the 2013 revision of the Declaration of Helsinki (https://www.wma.net/what-we-do/medical-ethics/declaration-of-helsinki/; accessed on 13 July 2017). All subjects gave their informed consent to participate in the study, which was approved by the clinical research ethics committees of Andalusia on 30 May 2017.

#### Data Analysis

A descriptive analysis was conducted, calculating the frequencies and percentages for qualitative variables and medians [P25–P75] for quantitative variables. The origin of HPs, their educational level, their relationship with partner and/or offspring, cause of homelessness, source of income, satisfaction with life, and happiness results were all treated as dichotomous variables to permit bivariate analyses, given the limited sample size. Fisher’s test was used to evaluate the relationship of happiness results with qualitative variables and the non-parametric Mann–Whitney test for their relationship with quantitative variables. Spearman’s rho test was applied to determine the relationships among quantitative variables. A *p* < 0.05 was considered significant. IBM Corp. Released 2010. IBM SPSS Statistics for Windows, Version 19.0. Armonk, NY, USA: IBM Corp was used for data analyses.

Qualitative analysis was conducted by a panel of three researchers following a descriptive content analysis procedure [24]. After reading the transcribed interviews, they encoded the most frequent responses and their number, organizing them into descriptive categories as a function of the main themes. Finally, the reliability of the design, data gathering, and data analysis was examined using a triangulation procedure with two external examiners for the encoding and identification of categories and themes [25,26]. The project was approved by the Ethics Committee of the Autonomous Community of Andalusia.

## 3. Results

Of the 30 HPs contacted for the initial quantitative phase of the study, 3 were excluded due to their condition at the scheduled time of the interview and 2 refused participation, leaving a final sample of 25 HPs. Of these 25 HPs, it was possible to make contact with 15 for the second phase of the study, which had a final sample of 14 HPs after excluding one participant for signs of mental confusion at the interview.

The median age of participants was 54 years, and 20 were males. They had lived on the street for a median of 0.9 [0.2–8.5] years, 13 (52.0%) had secondary or higher education, 20 (80.0%) had children, 9 (36.6%) attributed their homelessness to the economic crisis, 12 (48.0%) reported poor health, 15 (60.0%) had been assaulted on the street, 14 (56.0%) received no economic income, 12 (48.0%) were not seeking employment, 18 (72.0%) had not asked social services for assistance, 14 (56.0%) did not feel socially discriminated against, and 16 (64.0%) felt happy (Table 1).

There were no significant differences in any study variable between those who felt happy and those who felt unhappy (Table 2).

In the second phase of the study (n = 14), which included twelve men and two women, ten participants were classified with probable anxiety and twelve with probable depression; five reported being satisfied with life (grouping together the categories *highly satisfied, rather, and somewhat satisfied*); seven reported being happy (grouping together Happiness Scale categories *very happy and rather happy”*; and eleven had future expectations of improvement (Table 3).

A significant association was found between life satisfaction and happiness (*p* = 0.021). HPs who were satisfied with life were 4.5-fold more likely to feel happy than those who were not (OR 4.5). There was a close-to-significant tendency of higher anxiety among those who felt unhappy in comparison to those who felt happy (100.0% vs. 42.9%, *p* = 0.07). No association was found between expectations and happiness (*p* = 1) or between expectations and satisfaction with life (*p* = 0.72) (Table 4).

The sole significant correlation between quantitative variables was a negative correlation between the number of arrests and the satisfaction with life score, i.e., the more arrests, the lesser the satisfaction (rho = −0.655, *p* < 0.05).

### Analysis of Interviews

The analysis of interviews yielded three main themes that organized the discourses on happiness: conditions for happiness, their own happiness/unhappiness, and self-esteem (Table 5).

1. Theme: Conditions for happiness

We identified three categories that organized the conditions for happiness as indicated by participants: material, affective, and subjective (Table 5). Material conditions were described in terms of “*having*” objects, states, or situations, and were indicated by four participants. Affective conditions were emotions and affections related to “*love*” in the sense of giving, receiving, and sharing, indicated by four participants. Subjective conditions were personal conditions, attitudes, beliefs, and/or moods, indicated by eight participants (citations in Table 5).

2. Theme: Own happiness/unhappiness

This theme was related to different aspects related to their happiness/unhappiness articulated around “*feeling of current happiness*” and “*feeling of unhappiness*”. The former was expressed as their feelings at the time of the interview and what made them feel happy; six participants reported feeling happy and two partially happy. The reasons for feeling happy were mainly affective and subjective. The reasons for feeling unhappy were mainly related to subjective, emotional, and health aspects (citations in Table 5)

3. Theme: Self-esteem

This theme was related to self-satisfaction as an expression of inner coherence that can be part of the feeling of happiness. Discourses on this subject were generally brief and monosyllabic (in seven participants), contrasting with the more fulsome explanations on other issues. Responses to this theme were positive from nine participants, negative from three, and ambiguous from one (citations in Table 5).

## 4. Discussion

This study of HPs found an association between satisfaction with life and happiness, in agreement with various studies in the general population [27,28]. Satisfaction with life is a cognitive evaluation of the quality of one’s own experiences, an indicator of subjective wellbeing expressed by the individual [29]. The main components of subjective wellbeing are considered to be emotional responses or affections and satisfaction with life [21]. In their systemic review, Ngamaba et al. [30] concluded that life satisfaction is preferable to happiness as a measure of subjective well-being.

Future expectations were not related to happiness in this population (Table 4), contrasting with findings in the general Spanish population [31], although six participants in the qualitative study did relate happiness to both satisfaction with life and future expectations.

In the present sample of HPs, twelve were classified with probable depression and ten with probable anxiety, but neither was significantly associated with happiness/unhappiness; however, there was a tendency towards an association with probable anxiety, which has been reported in the general population [32,33], and the failure to reach significance may be attributable to the small sample size. We cannot rule out the effect of other psychopathologies on the self-perceived happiness/unhappiness of HPs, and further research is required on this issue.

The study by Cruz-Teran et al. [34] in Granada found a higher percentage of HPs who were satisfied with their lives. Besides differences in study design and measurement instruments, their survey took place before the economic crisis suffered by Spain and other European studies, which may contribute to this discrepancy. The authors concluded that the HPs in their study had little awareness of the reality in which they were immersed and denied their reality to strangers and those not living on the street as a survival strategy.

No significant association was found between the responses to the question “*Do you feel happy?*” and any of the independent variables studied. Feeling happy was not related to age, sex, educational level, or housing situation, in agreement with the study by Panadero et al. [15] of 235 HPs. However, unlike this previous report, we found no association with a positive self-perception of health status in the first phase or future expectations in the quantitative study in the second phase, and health did not emerge as a happiness-conditioning theme in the qualitative study, although future expectations did, in various ways. These differences with the study by Panadero et al. [15] may be explained not only by the subjectivity of the participants’ perception of their own health, but also by the different manner in which this information was gathered and the small sample size. Panadero et al. [15] reported a much more positive self-perception of health among those who felt happy rather than unhappy, and greater happiness was associated with a lesser degree of disability or severe/chronic disease, using a variety of questions and response categories. In contrast, the present participants responded to a single question on health with only two options (good or poor health).

Panadero and coworkers [15] also described an association of happiness with religious belief in HPs, as in other populations [32,35], and HPs were found to score significantly higher for religious and spiritual beliefs in comparison to people with homes [7]. This aspect was not evaluated in our quantitative study and was not raised as a major theme in the interviews with HPs.

Panadero et al. [15] found that happiness was not associated with having partners, family or friends, although the HPs felt happier “*if they felt less abandoned*”. We did not gather data on this variable as such, but analogous expressions were frequent in the interviews, including: “*being happy with the family*”, “*seeing family and friends*”, “*happiness of the group*”, and “*happiness of others*”. The qualitative results obtained in the present study are in agreement with the conclusion of other studies [13,36] that happiness, life satisfaction, and subjective well-being are highly influenced by personal relationships (e.g., family, friends, acquaintances) and are associated with a concern for others, in line with previous findings in HPs [16,37].

Regarding self-esteem, more than half of the HPs in the qualitative study expressed feelings of satisfaction with themselves, similar to the finding by Cruz Teran et al. [33] that HPs generally have a rather optimistic perception of themselves; however, a minority of participants linked happiness to *“having what is normal*”, “*having a good lifestyle, a house, a job*…”.

The high percentage of HPs who reported feeling happy and satisfied offered various explanations for these states. Variables that were not considered in our study may also be relevant. For instance, a biological approach to happiness is gathering strength, and Panadero et al. [15] discussed the idea that optimism is a personality characteristic. Future research will shed light on the biological foundations of happiness and their implications, and a biological approach to positive emotions [38] and altruism [39] will also be taken into account.

The HPs in our study did not tend to seek assistance from social services, despite generally being without unemployment, sleeping on the street, suffering assaults, and having a poor relationship with their families. These findings may help community nurses to better understand the alienation and mistrust felt by this stigmatized group and improve their approach to delivering care. Knowledge of what makes people in this situation feel happy and satisfied could be useful in the design of interventions to improve the health and well-being of the homeless. As noted by Vázquez-Souza [40], programs should be adapted to the places where HPs can be found and flexible to provide the continuous care that is required, including the treatment of disabilities, despair, fear, loneliness, and low self-esteem.

Finally, there were much fewer women than men, as in other studies of this type [41], suggesting that women may preferentially seek informal solutions from friends and family members, contacting social services as a last resort. In addition, social services usually prioritize the provision of shelter to women sleeping on the street because of their frequent exposure to violence and sexual assault [42].

The large number of HPs feeling satisfied with their life and happy might suggest that many have a strong capacity for life adjustment, among other qualities, although this can only be considered as a speculative conclusion.

## 5. Conclusions

HPs who felt satisfied with life were 4.5-fold more likely to feel happy than those who did not. Happiness was related to material aspects, affective situations, and daily life activities, which they valued but did not possess. The majority of HPs felt good about themselves and half of them felt happy, despite their adverse situations, which included poor family relationships, no income, weak health, sleeping on the street, and vulnerability to assault and arrest. Knowledge of the feelings of HPs can help community nurses and other social care providers to approach this vulnerable population and can assist in the design of effective public health interventions to improve their physical and mental health.

### Study Limitations

It is especially challenging to obtain honest responses to highly personal questions from this very fragile population. In order to minimize this potential limitation, the researchers spent two years establishing connections with HPs in the city on the street and in the *Calor y Café* center. Participants were selected by intentional sampling and only included HPs that were willing to participate and take part in a recorded interview. In addition, the results cannot be generalized due to the small sample size and the exploratory nature of the study, limiting the conclusions that can be drawn. A major obstacle proved to be the difficulty in contacting participants from the first phase of the study to take part in the second. Finally, further research is warranted to explore the impact of psychopathology on HPs’ self-perceived health.

## Figures and Tables

**Table 1:** Characteristics of the 25 participants.

Variable	Median [P25–P75] or n (%)
Age (years)	54.0 [46.0–58.0]
Years on the street	0.9 [0.2–8.5]
Children	2.0 [1.0–3.0]
Arrested	2.0 [0.0–4.0]
Sex (male)	20 (80.0)
Origin	
Granada	11 (44.0)
Rest of Andalusia	7 (28.0)
Rest of Spain	3 (12.0)
Foreigners	4 (16.0)
Educational status	
No studies/primary school	12 (48.0)
Secondary school	8 (32.0)
Professional training	3 (12.0)
University degree	2 (8.0)
Homeless causes	
Economic crisis	9 (36.0)
Divorce	6 (24.0)
Family conflicts	7 (28.0)
Other causes	3 (12.0)
Health (good/poor)	
Reports poor health	12 (48.0)
Reports good health	13 (52.0)
Has been assaulted (yes/no)	
Yes	15 (60.0)
No	10 (40.0)
Sleeps	
On the street	16 (64.0)
In temporary lodgings	9 (36.0)
Source of income	
No income	14 (56.0)
Non-contributory pension	10 (40.0)
Temporary employment	1 (4.0)
Jobseeker (yes/no)	
Yes	12 (48.0)
No	13 (52.0)
Requested assistance from social services (yes/no)	
Yes	7 (28.0)
No	18 (72.0)
Feel discriminated against (yes/no)	
Yes	11 (44.0)
No	14 (56.0)
Feel happy (yes/no)	
Yes	16 (64.0)
No	9 (36.0)
Median [P25–P75]: Median [interquartile range]	

**Table 2:** Association of the question “Do you feel happy?” (yes/no) with other study variables.

Variable	Happy (n = 16)	Unhappy (n = 9)	*p*
	n (%)	n (%)	
Sex (male)	12 (75.0)	8 (88.9)	0.621
Origin (Granada)	9 (56.3)	2 (22.2)	0.208
Educational level (primary studies)	10 (62.5)	2 (22.2)	0.097
Causes (family conflicts)	8 (50.0)	5 (55.6)	1.000
Perceived health (good)	8 (50.0)	5 (55.6)	1.000
Assaulted (yes)	11 (68.8)	4 (44.4)	0.397
Sleeps (street)	9 (56.3)	7 (77.8)	0.401
Economic income (no)	10 (62.5)	4 (44.4)	0.434
Jobseeker (no)	10 (62.5)	3 (33.3)	0.226
Assistance requested (no)	11 (68.8)	7 (77.8)	1.000
Perceived discrimination (no)	11 (68.8)	6 (66.7)	0.115
	Median [P25–P75]		
Age	54.0 [41.5–56.5]	54.0 [48.3–58.0]	0.329
Years on the street	0.6 [0.2–20.0]	1.0 [0.1–8.0]	0.519
N° times arrested	3.0 [0.0–4.0]	2.0 [0 0–4.0]	0.890
N° children	2.0 [0.0–3.0]	2.0 [1.3–3.0]	0.452
Median [P25–P75]: Median [interquartile range]			

**Table 3:** Descriptive analysis of the scales of anxiety, depression, satisfaction with life, happiness, and responses on future expectations.

Scale	Median [P25–P75] or n (%) n = 14
Index Golberg anxiety subscale	5.5 [3.3–7.0]
Probable anxiety (yes/no)	
Yes	10 (71.4)
No	4 (28.6)
Index Golberg depression subscale	4.0 [3.0–5.0]
Probable depression (yes/no)	
Yes	12 (85.7)
No	2 (14.3)
Satisfaction with life	
Yes	5 (35.7)
No	9 (64.3)
Happiness	
Yes	7 (50.0)
No	7 (50.0)
Expectations	
Improvement	11 (78.6)
Remaining the same	3 (21.4)
Worsening	0 (0.0)
Median [P25–P75]: Median [interquartile range]	

**Table 4:** Bivariate analysis of the variable happy/unhappy on the happiness scale with respect to the anxiety/depression scale, the satisfaction with life, and the response on future expectations.

	Happy (n = 7)	Unhappy (n = 7)	*p*
Probable anxiety (yes)	3 (42.9)	7 (100.0)	0.070
Probable depression (yes)	5 (71.4)	7 (100.0)	0.462
Satisfaction with life (satisfied)	5 (71.4)	0 (0.0)	0.021
Future expectations (improvement)	7 (71.4)	6 (85.7)	1

**Table 5:** Themes, categories, and textual citations of the interviews.

Theme	Categories	Citations
Conditions for happiness	Material (n = 4)	“…it’s that you don’t lack anything, being happy with your family, having a good lifestyle, having a house; having a job” (P01, male)“…having your job, your house, and those things. What’s normal, no?” (P02, male)
	Affective (n = 4)	“Happiness is someone, a person who supports you, who loves you.” (P07, female) “For me, happiness is love, affection, and everything you can give someone. Give to someone even though you don’t receive anything in exchange… It consists in giving inner love from your body, from your soul, that you give it to the people you know.” (P11, male)
	Subjective (n = 8)	“…happiness is getting up in the morning and not thinking I have to do this or the other... I mean, relaxation…, happiness can be the ability to say “no”, it’s looking forward and not seeing so many obstacles” (P04, male) “Happiness for me... is to be right with God and to see your family, your friends, and... It doesn’t depend on you, on us... I mean it.” (P05 male) “A moment of happiness can be a plate of food you’ve cooked, a smile from your children… a look because you’ve connected and got feedback or a feeling with the person you’re interacting with.” (P06 male) “Well, having a good time, being comfortable, it’s to go… go dancing that I love.” (P10 male)
Own happiness/unhappiness	Current feeling of happiness (n = 6 happy) (n = 2 partially happy)	“happy? …from time to time, from time to time. [It makes me happy] Being with friends. Playing cards for example, it’s not one thing in particular.” (P02 male) “Very happy… It’s just that right now I’m with my partner, and I hadn’t seen my daughter for five years and I saw her on the 5th, the day of the Epiphany” (P07, female) “Very happy… Anything makes me happy, even the air makes me happy, and being alive every day, that makes me happy.” (P08 male)
	Feeling of unhappiness (n = 6)	“Very unhappy. What’s made me happy all my life has been the happiness of others, the happiness of the group. Being with people who you connect with … If I had the chance of starting again…” (P06 male) “Unhappy. Today I feel rather depressed and rather bad because of the injustices you suffer. The things that put me off are the bad friendships you have by your side who betray you, and deceive you, and rip you off, and take away what you have...” (P10 male)
Self-esteem	Feeling of satisfaction with themselves (n = 9 affirmative) (n = 3 negative) (n = 1 ambiguous)	“No… I don’t feel good about myself” (P01 male) “Me? About myself? Of course.” (P03 male) “Eh… I don’t think so… On the one hand yes and on the other no. Yes, because I can die in peace.” (P06 male) “It’s that I never feel good about myself” (P09, female) “I do feel good about myself and I’m proud of what I’m doing.” (P10 male)

## Data Availability

The underlying data are available on request.
